# New Imaging in Gastrointestinal Tract

**DOI:** 10.1155/2016/5785871

**Published:** 2016-02-04

**Authors:** Roberto Grassi, Antonio Pinto, Lorenzo Mannelli, Daniele Marin, Maria Antonietta Mazzei

**Affiliations:** ^1^Institute of Radiology, Second University of Naples, 80138 Naples, Italy; ^2^Department of Radiology, Cardarelli Hospital, 80123 Naples, Italy; ^3^Department of Radiology, Memorial Sloan Kettering Cancer Center, 1275 York Avenue, New York, NY 10065, USA; ^4^Department of Radiology, Duke University Medical Center, Durham, NC 27710, USA; ^5^Department of Medical, Surgical and Neuro Sciences, Diagnostic Imaging, University of Siena, Azienda Ospedaliera Universitaria Senese, Viale Bracci 10, 53100 Siena, Italy

Pathologies of gastrointestinal tract are various and affect patients of different ages. Both of these conditions influence the imaging modalities of gastrointestinal tract that underwent relevant changes during recent years. Magnetic Resonance (MR) and Computed Tomography (CT) techniques, optimised for gastrointestinal imaging, are playing today an increasing role in the evaluation of gastrointestinal disorders, and several studies have shown the advantage of these techniques over tradition barium fluoroscopic examinations secondary to improvements in spatial and temporal resolution combined with improved bowel distending agents. Based on recent literature and guidelines, there is a change of paradigms regarding the diagnosis of esophagus and gastrointestinal cancer towards CT, whereas for small bowel imaging in inflammatory disease MRI with a new focus on Diffusion Weighted Imaging (DWI) are the most important imaging modalities, because DWI can be easily implemented in standard MRI for routine use to further enhance the diagnostic accuracy in disease assessment [1–4]. CT and MRI play an important role also in functional disorders. In particular, the recent development of faster MRI pulse sequences provides rapid, real-time imaging of the gastrointestinal tract, pinpointing areas of stricture and providing valuable information on motility.

This special issue is devoted to current and emerging techniques in gastrointestinal tract, focusing on some selected topics that are both interesting and challenging: neoplastic pathologies, chronic inflammatory diseases, functional pathologies, and nontraumatic emergency causing occlusion. The first section covers cross-sectional imaging of the gastrointestinal tract in neoplastic disease, including lymphoma, both through a review (“Radiological Features of Gastrointestinal Lymphoma” by G. Lo Re et al.) and through an original paper (“Staging of Primary Abdominal Lymphomas: Comparison of Whole-Body MRI with Diffusion-Weighted Imaging and ^18^F-FDG-PET/CT” by A. Stecco et al.) and small-bowel neoplasms (“Small-Bowel Neoplasms: Role of MRI Enteroclysis” by A. Faggian et al.). The imaging of hepatocellular carcinoma after locoregional treatments is also reviewed (“CT Appearance of Hepatocellular Carcinoma after Locoregional Treatments: A Comprehensive Review” by D. Marin et al.). Cross-sectional imaging modalities are fundamental also in the management of patients with inflammatory bowel disease (IBD) from the first diagnosis and throughout the entire course of the disease. In this sense, MRI, owing to the lack of ionizing radiation, represents the main technique in young patients with IBD who may require multiple studies over a lifetime. New imaging of chronic inflammatory pathologies is focused on Crohn's disease, where the imaging is essential also in scoring the activity of disease (“3D-EAUS and MRI in the Activity of Anal Fistulas in Crohn's Disease” by M. E. Alabiso et al.; “Assessment of Disease Activity in Small Bowel Crohn's Disease: Comparison between Endoscopy and Magnetic Resonance Enterography Using Mria and Modified Mria Score” by A. Scardapane et al.). Some functional pathologies are also discussed: achalasia and pelvic floor disfunction (“Imaging in the Evaluation of Endoscopic or Surgical Treatment for Achalasia” by D. Palladino et al.; “MR Imaging in Diagnosis of Pelvic Floor Descent: Supine versus Sitting Position” by F. Iacobellis et al.). Finally nontraumatic emergency causing occlusion is discussed in three different papers, with emphasis on the role of MDCT and dynamic MRI (“Intussusception in Adults: The Role of MDCT in the Identification of the Site and Cause of Obstruction” by V. Valentini et al.; “A Novel Diagnostic Aid for Detection of Intra-Abdominal Adhesions to the Anterior Abdominal Wall Using Dynamic Magnetic Resonance Imaging” by D. Randall et al.; “Adhesions to Mesh after Ventral Hernia Mesh Repair are Detected by MRI but Are Not a Cause of Long Term Chronic Abdominal Pain” by O. Langbach et al.).

The contributions of this special issue could stimulate the spread of new imaging modalities in daily practice, pinpoint technical aspects, and share some strategies to optimise CT and MR protocols.

*Roberto Grassi* *Roberto Grassi* *Antonio Pinto* *Antonio Pinto* *Lorenzo Mannelli* *Lorenzo Mannelli* *Daniele Marin* *Daniele Marin* *Maria Antonietta Mazzei* *Maria Antonietta Mazzei*
